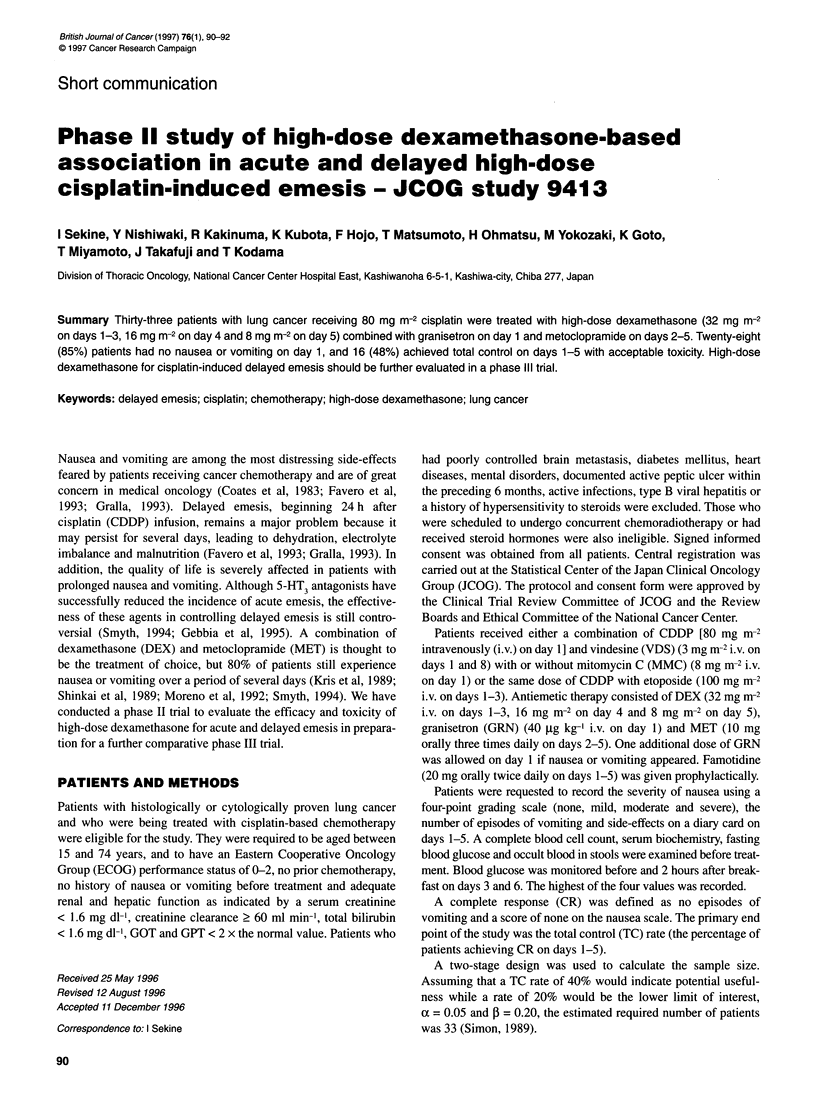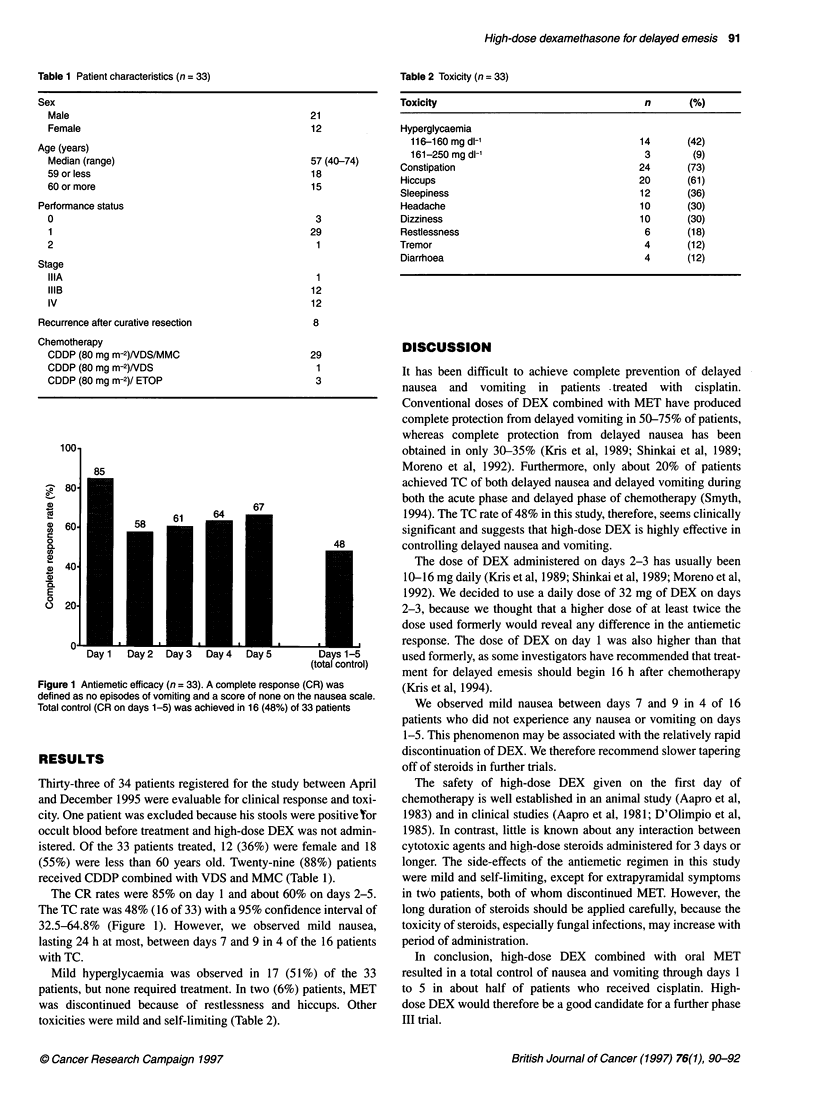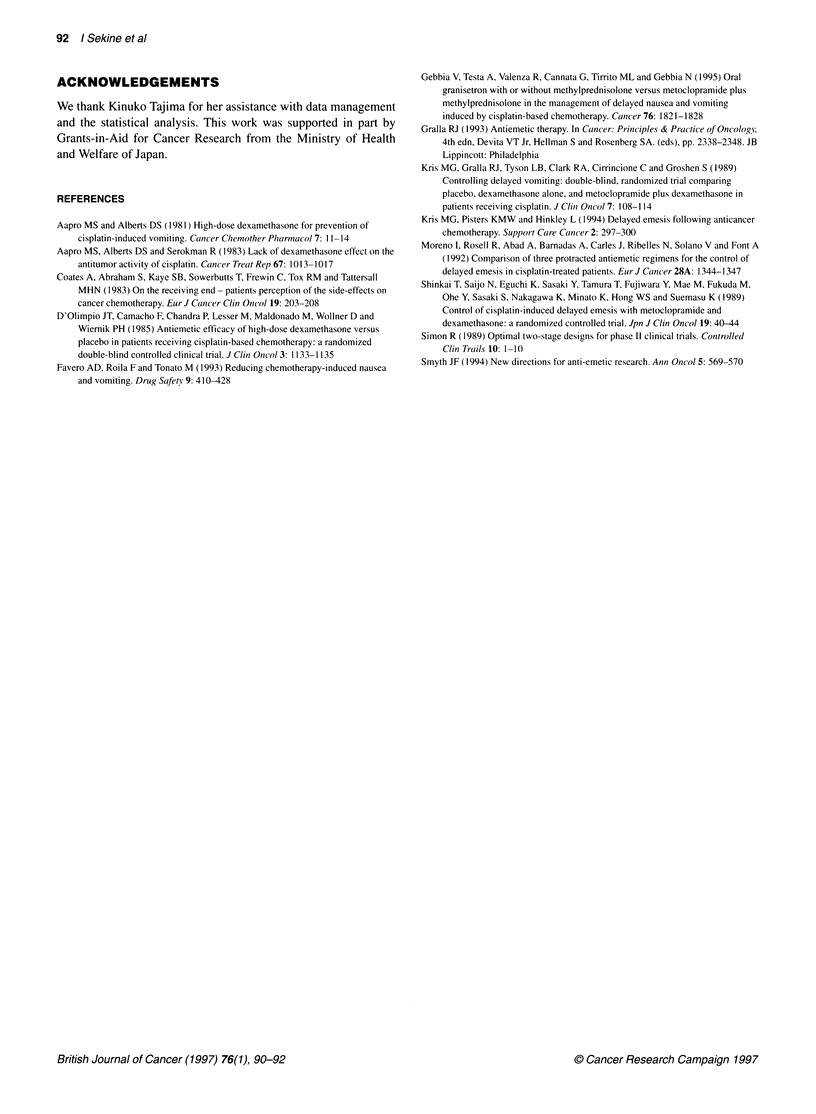# Phase II study of high-dose dexamethasone-based association in acute and delayed high-dose cisplatin-induced emesis--JCOG study 9413.

**DOI:** 10.1038/bjc.1997.341

**Published:** 1997

**Authors:** I. Sekine, Y. Nishiwaki, R. Kakinuma, K. Kubota, F. Hojo, T. Matsumoto, H. Ohmatsu, M. Yokozaki, K. Goto, T. Miyamoto, J. Takafuji, T. Kodama

**Affiliations:** Division of Thoracic Oncology, National Cancer Center Hospital East, Kashiwa-city, Chiba, Japan.

## Abstract

Thirty-three patients with lung cancer receiving 80 mg m(-2) cisplatin were treated with high-dose dexamethasone (32 mg m(-2) on days 1-3, 16 mg m(-2) on day 4 and 8 mg m(-2) on day 5) combined with granisetron on day 1 and metoclopramide on days 2-5. Twenty-eight (85%) patients had no nausea or vomiting on day 1, and 16 (48%) achieved total control on days 1-5 with acceptable toxicity. High-dose dexamethasone for cisplatin-induced delayed emesis should be further evaluated in a phase III trial.


British Journal of Cancer (1997) 76(1), 90–92
© 1997 Cancer Research Campaign

Short communication

### Phase II study of high-dose dexamethasone-based association in acute and delayed high-dose cisplatin-induced emesis – JCOG study 9413

**I Sekine, Y Nishiwaki, R Kakinuma, K Kubota, F Hojo, T Matsumoto, H Ohmatsu, M Yokozaki, K Goto, T Miyamoto, J Takafuji and T Kodama**

Division of Thoracic Oncology, National Cancer Center Hospital East, Kashiwanoha 6-5-1, Kashiwa-city, Chiba 277, Japan

**Summary** Thirty-three patients with lung cancer receiving 80 mg $m^{-2}$ cisplatin were treated with high-dose dexamethasone (32 mg $m^{-2}$ on days 1–3, 16 mg $m^{-2}$ on day 4 and 8 mg $m^{-2}$ on day 5) combined with granisetron on day 1 and metoclopramide on days 2–5. Twenty-eight (85%) patients had no nausea or vomiting on day 1, and 16 (48%) achieved total control on days 1–5 with acceptable toxicity. High-dose dexamethasone for cisplatin-induced delayed emesis should be further evaluated in a phase III trial.

**Keywords:** delayed emesis; cisplatin; chemotherapy; high-dose dexamethasone; lung cancer

Nausea and vomiting are among the most distressing side-effects feared by patients receiving cancer chemotherapy and are of great concern in medical oncology (Coates et al, 1983; Favero et al, 1993; Gralla, 1993). Delayed emesis, beginning 24 h after cisplatin (CDDP) infusion, remains a major problem because it may persist for several days, leading to dehydration, electrolyte imbalance and malnutrition (Favero et al, 1993; Gralla, 1993). In addition, the quality of life is severely affected in patients with prolonged nausea and vomiting. Although 5-$HT_3$ antagonists have successfully reduced the incidence of acute emesis, the effectiveness of these agents in controlling delayed emesis is still controversial (Smyth, 1994; Gebbia et al, 1995). A combination of dexamethasone (DEX) and metoclopramide (MET) is thought to be the treatment of choice, but 80% of patients still experience nausea or vomiting over a period of several days (Kris et al, 1989; Shinkai et al, 1989; Moreno et al, 1992; Smyth, 1994). We have conducted a phase II trial to evaluate the efficacy and toxicity of high-dose dexamethasone for acute and delayed emesis in preparation for a further comparative phase III trial.

### PATIENTS AND METHODS

Patients with histologically or cytologically proven lung cancer and who were being treated with cisplatin-based chemotherapy were eligible for the study. They were required to be aged between 15 and 74 years, and to have an Eastern Cooperative Oncology Group (ECOG) performance status of 0–2, no prior chemotherapy, no history of nausea or vomiting before treatment and adequate renal and hepatic function as indicated by a serum creatinine < 1.6 mg $dl^{-1}$, creatinine clearance ≥ 60 ml $min^{-1}$, total bilirubin < 1.6 mg $dl^{-1}$, GOT and GPT < 2 × the normal value. Patients who had poorly controlled brain metastasis, diabetes mellitus, heart diseases, mental disorders, documented active peptic ulcer within the preceding 6 months, active infections, type B viral hepatitis or a history of hypersensitivity to steroids were excluded. Those who were scheduled to undergo concurrent chemoradiotherapy or had received steroid hormones were also ineligible. Signed informed consent was obtained from all patients. Central registration was carried out at the Statistical Center of the Japan Clinical Oncology Group (JCOG). The protocol and consent form were approved by the Clinical Trial Review Committee of JCOG and the Review Boards and Ethical Committee of the National Cancer Center.

Patients received either a combination of CDDP [80 mg $m^{-2}$ intravenously (i.v.) on day 1] and vindesine (VDS) (3 mg $m^{-2}$ i.v. on days 1 and 8) with or without mitomycin C (MMC) (8 mg $m^{-2}$ i.v. on day 1) or the same dose of CDDP with etoposide (100 mg $m^{-2}$ i.v. on days 1–3). Antiemetic therapy consisted of DEX (32 mg $m^{-2}$ i.v. on days 1–3, 16 mg $m^{-2}$ on day 4 and 8 mg $m^{-2}$ on day 5), granisetron (GRN) (40 μg $kg^{-1}$ i.v. on day 1) and MET (10 mg orally three times daily on days 2–5). One additional dose of GRN was allowed on day 1 if nausea or vomiting appeared. Famotidine (20 mg orally twice daily on days 1–5) was given prophylactically.

Patients were requested to record the severity of nausea using a four-point grading scale (none, mild, moderate and severe), the number of episodes of vomiting and side-effects on a diary card on days 1–5. A complete blood cell count, serum biochemistry, fasting blood glucose and occult blood in stools were examined before treatment. Blood glucose was monitored before and 2 hours after breakfast on days 3 and 6. The highest of the four values was recorded.

A complete response (CR) was defined as no episodes of vomiting and a score of none on the nausea scale. The primary end point of the study was the total control (TC) rate (the percentage of patients achieving CR on days 1–5).

A two-stage design was used to calculate the sample size. Assuming that a TC rate of 40% would indicate potential usefulness while a rate of 20% would be the lower limit of interest, $\alpha = 0.05$ and $\beta = 0.20$, the estimated required number of patients was 33 (Simon, 1989).

*Received 25 May 1996*
*Revised 12 August 1996*
*Accepted 11 December 1996*

*Correspondence to:* I Sekine

**Table 1** Patient characteristics ($n$ = 33)

| | |
|---|---|
| Sex | |
| Male | 21 |
| Female | 12 |
| Age (years) | |
| Median (range) | 57 (40–74) |
| 59 or less | 18 |
| 60 or more | 15 |
| Performance status | |
| 0 | 3 |
| 1 | 29 |
| 2 | 1 |
| Stage | |
| IIIA | 1 |
| IIIB | 12 |
| IV | 12 |
| Recurrence after curative resection | 8 |
| Chemotherapy | |
| CDDP (80 mg m$^{-2}$)/VDS/MMC | 29 |
| CDDP (80 mg m$^{-2}$)/VDS | 1 |
| CDDP (80 mg m$^{-2}$)/ ETOP | 3 |

**Figure 1** Antiemetic efficacy ($n$ = 33). A complete response (CR) was defined as no episodes of vomiting and a score of none on the nausea scale. Total control (CR on days 1–5) was achieved in 16 (48%) of 33 patients

### RESULTS

Thirty-three of 34 patients registered for the study between April and December 1995 were evaluable for clinical response and toxicity. One patient was excluded because his stools were positive for occult blood before treatment and high-dose DEX was not administered. Of the 33 patients treated, 12 (36%) were female and 18 (55%) were less than 60 years old. Twenty-nine (88%) patients received CDDP combined with VDS and MMC (Table 1).

The CR rates were 85% on day 1 and about 60% on days 2–5. The TC rate was 48% (16 of 33) with a 95% confidence interval of 32.5–64.8% (Figure 1). However, we observed mild nausea, lasting 24 h at most, between days 7 and 9 in 4 of the 16 patients with TC.

Mild hyperglycaemia was observed in 17 (51%) of the 33 patients, but none required treatment. In two (6%) patients, MET was discontinued because of restlessness and hiccups. Other toxicities were mild and self-limiting (Table 2).

**Table 2** Toxicity ($n$ = 33)

| Toxicity | $n$ | (%) |
|---|---|---|
| Hyperglycaemia | | |
| 116–160 mg dl$^{-1}$ | 14 | (42) |
| 161–250 mg dl$^{-1}$ | 3 | (9) |
| Constipation | 24 | (73) |
| Hiccups | 20 | (61) |
| Sleepiness | 12 | (36) |
| Headache | 10 | (30) |
| Dizziness | 10 | (30) |
| Restlessness | 6 | (18) |
| Tremor | 4 | (12) |
| Diarrhoea | 4 | (12) |

### DISCUSSION

It has been difficult to achieve complete prevention of delayed nausea and vomiting in patients treated with cisplatin. Conventional doses of DEX combined with MET have produced complete protection from delayed vomiting in 50–75% of patients, whereas complete protection from delayed nausea has been obtained in only 30–35% (Kris et al, 1989; Shinkai et al, 1989; Moreno et al, 1992). Furthermore, only about 20% of patients achieved TC of both delayed nausea and delayed vomiting during both the acute phase and delayed phase of chemotherapy (Smyth, 1994). The TC rate of 48% in this study, therefore, seems clinically significant and suggests that high-dose DEX is highly effective in controlling delayed nausea and vomiting.

The dose of DEX administered on days 2–3 has usually been 10–16 mg daily (Kris et al, 1989; Shinkai et al, 1989; Moreno et al, 1992). We decided to use a daily dose of 32 mg of DEX on days 2–3, because we thought that a higher dose of at least twice the dose used formerly would reveal any difference in the antiemetic response. The dose of DEX on day 1 was also higher than that used formerly, as some investigators have recommended that treatment for delayed emesis should begin 16 h after chemotherapy (Kris et al, 1994).

We observed mild nausea between days 7 and 9 in 4 of 16 patients who did not experience any nausea or vomiting on days 1–5. This phenomenon may be associated with the relatively rapid discontinuation of DEX. We therefore recommend slower tapering off of steroids in further trials.

The safety of high-dose DEX given on the first day of chemotherapy is well established in an animal study (Aapro et al, 1983) and in clinical studies (Aapro et al, 1981; D'Olimpio et al, 1985). In contrast, little is known about any interaction between cytotoxic agents and high-dose steroids administered for 3 days or longer. The side-effects of the antiemetic regimen in this study were mild and self-limiting, except for extrapyramidal symptoms in two patients, both of whom discontinued MET. However, the long duration of steroids should be applied carefully, because the toxicity of steroids, especially fungal infections, may increase with period of administration.

In conclusion, high-dose DEX combined with oral MET resulted in a total control of nausea and vomiting through days 1 to 5 in about half of patients who received cisplatin. High-dose DEX would therefore be a good candidate for a further phase III trial.

© Cancer Research Campaign 1997 

### ACKNOWLEDGEMENTS

We thank Kinuko Tajima for her assistance with data management and the statistical analysis. This work was supported in part by Grants-in-Aid for Cancer Research from the Ministry of Health and Welfare of Japan.

### REFERENCES

Aapro MS and Alberts DS (1981) High-dose dexamethasone for prevention of cisplatin-induced vomiting. *Cancer Chemother Pharmacol* **7**: 11–14

Aapro MS, Alberts DS and Serokman R (1983) Lack of dexamethasone effect on the antitumor activity of cisplatin. *Cancer Treat Rep* **67**: 1013–1017

Coates A, Abraham S, Kaye SB, Sowerbutts T, Frewin C, Tox RM and Tattersall MHN (1983) On the receiving end – patients perception of the side-effects on cancer chemotherapy. *Eur J Cancer Clin Oncol* **19**: 203–208

D'Olimpio JT, Camacho F, Chandra P, Lesser M, Maldonado M, Wollner D and Wiernik PH (1985) Antiemetic efficacy of high-dose dexamethasone versus placebo in patients receiving cisplatin-based chemotherapy: a randomized double-blind controlled clinical trial. *J Clin Oncol* **3**: 1133–1135

Favero AD, Roila F and Tonato M (1993) Reducing chemotherapy-induced nausea and vomiting. *Drug Safety* **9**: 410–428

Gebbia V, Testa A, Valenza R, Cannata G, Tirrito ML and Gebbia N (1995) Oral granisetron with or without methylprednisolone versus metoclopramide plus methylprednisolone in the management of delayed nausea and vomiting induced by cisplatin-based chemotherapy. *Cancer* **76**: 1821–1828

Gralla RJ (1993) Antiemetic therapy. In *Cancer: Principles & Practice of Oncology*, 4th edn, Devita VT Jr, Hellman S and Rosenberg SA. (eds), pp. 2338–2348. JB Lippincott: Philadelphia

Kris MG, Gralla RJ, Tyson LB, Clark RA, Cirrincione C and Groshen S (1989) Controlling delayed vomiting: double-blind, randomized trial comparing placebo, dexamethasone alone, and metoclopramide plus dexamethasone in patients receiving cisplatin. *J Clin Oncol* **7**: 108–114

Kris MG, Pisters KMW and Hinkley L (1994) Delayed emesis following anticancer chemotherapy. *Support Care Cancer* **2**: 297–300

Moreno I, Rosell R, Abad A, Barnadas A, Carles J, Ribelles N, Solano V and Font A (1992) Comparison of three protracted antiemetic regimens for the control of delayed emesis in cisplatin-treated patients. *Eur J Cancer* **28A**: 1344–1347

Shinkai T, Saijo N, Eguchi K, Sasaki Y, Tamura T, Fujiwara Y, Mae M, Fukuda M, Ohe Y, Sasaki S, Nakagawa K, Minato K, Hong WS and Suemasu K (1989) Control of cisplatin-induced delayed emesis with metoclopramide and dexamethasone: a randomized controlled trial. *Jpn J Clin Oncol* **19**: 40–44

Simon R (1989) Optimal two-stage designs for phase II clinical trials. *Controlled Clin Trails* **10**: 1–10

Smyth JF (1994) New directions for anti-emetic research. *Ann Oncol* **5**: 569–570

© Cancer Research Campaign 1997